# Spontaneous intracranial hypotension: diagnostic and therapeutic workup

**DOI:** 10.1007/s00234-021-02766-z

**Published:** 2021-07-23

**Authors:** Niklas Luetzen, Philippe Dovi-Akue, Christian Fung, Juergen Beck, Horst Urbach

**Affiliations:** 1 Department of Neuroradiology, Medical Center, University of Freiburg, Breisacher Str. 64 , 79106 Freiburg, Germany; 2 Department of Neurosurgery, Medical Center, University of Freiburg, Freiburg, Germany

**Keywords:** Spontaneous intracranial hypotension, CSF-venous fistula, Lateral decubitus myelography

## Abstract

Spontaneous intracranial hypotension (SIH) is an orthostatic headache syndrome with typical MRI findings among which engorgement of the venous sinuses, pachymeningeal enhancement, and effacement of the suprasellar cistern have the highest diagnostic sensitivity. SIH is in almost all cases caused by spinal CSF leaks. Spinal MRI scans showing so-called spinal longitudinal extradural fluid (SLEC) are suggestive of ventral dural tears (type 1 leak) which are located with prone dynamic (digital subtraction) myelography. As around half of the ventral dural tears are located in the upper thoracic spine, additional prone dynamic CT myelography is often needed. Leaking nerve root sleeves typically associated with meningeal diverticulae (type 2 leaks) and CSF-venous fistulas (type 3 leaks) are proven via lateral decubitus dynamic digital subtraction or CT myelography: type 2 leaks are SLEC-positive if the tear is proximal and SLEC-negative if it is distal, and type 3 leaks are always SLEC-negative. Although 30–70% of SIH patients show marked improvement following epidural blood patches applied via various techniques definite cure mostly requires surgical closure of ventral dural tears and surgical ligations of leaking nerve root sleeves associated with meningeal diverticulae or CSF-venous fistulas. For the latter, transvenous embolization with liquid embolic agents via the azygos vein system is a novel and valuable therapeutic alternative.

Spontaneous intracranial hypotension (SIH) is an orthostatic headache syndrome with an estimated annual incidence of 5/100.00 [1, 2]. It is pathogenetically separated from that of postdural puncture headache and from postoperative cerebrospinal fluid (CSF) loss [1]. Females are more often affected than males (2:1), the peak incidence is around 40 years of age, and SIH is rare but not absent in children [3–6]. The key symptom is orthostatic headache which generally occurs or worsens within 15 min of assuming the upright position and which tends to increase in severity over the course of the day. Most patients can clearly identify the day when the headache and other symptoms began [7, 8]. The headache is most pronounced in the back of the head, which can be explained as the result of the following causal sequence: low CSF volume—sagging of the brain—tension on the cranial nerves and dura mater. The dura mater is especially tension-sensitive in the posterior fossa [7]. In patients who have been suffering from SIH for longer periods—the average latency from symptom onset to diagnosis is 3 months—the headaches can lose their positional dependence, or even worsen when the patient reclines [7–9]. About half of all patients complain of auditory disturbances (“ringing in the ears,” tinnitus, a pressure sensation in the ear), and some are initially treated for sudden hearing loss or suspected Ménière’s disease [10]. SIH can have a wide range of further presentations, ranging all the way to coma or apparent frontotemporal dementia [11–14].

## Diagnostic workup

Diagnostic criteria include a CSF pressure < 60 mm H_2_O and/or evidence of a CSF leak on imaging [1]. As lumbar puncture is invasive and only one-third of SIH patients has a CSF opening pressure < 60 mm H_2_O, MRI of the head and the spine is mandatory [15]. Note that particularly in patients with a long history of SIH or with large abdominal girth CSF opening pressure is often normal or even elevated [15–17].

## Head MRI

Numerous cranial MRI signs of SIH have been described using different MRI sequences on 1.5 and 3 Tesla scanners. There is neither a definite MRI sign nor a definite MRI protocol, and it is rather the combination of MRI signs that allow to diagnose SIH with a high grade of certainty [12]. Dobrocky et al. proposed a score for the most accurate MRI signs in 2019 which has been later termed the Bern score [18, 19]. Pachymeningeal contrast enhancement, engorgement of the venous sinuses and effacement of the suprasellar cistern of 4.0 mm or less were shown to be the most important discriminating features between SIH patients and normal controls and weighted with 2 points each. Subdural fluid collections, effacement of the prepontine cistern of 5 mm or less, and a mamillopontine distance of 6.5 mm or less were weighted with 1 point each [18]. Patients with total scores of 2 points or fewer were classified as having a low, with 3 to 4 points as having an intermediate, and with 5 or more points as having a high probability for a spinal CSF leak, which is in almost all cases the underlying cause of SIH (Table 1) [18–21]. Engorgement of the venous sinuses does not only mean an increased volume but also a change of shape with, e.g., the inferior margin of the midportion of the dominant transverse sinus showing a distended convex appearance called the venous distension sign [22]. Some of the MRI signs are somewhat arbitrary in definition which can lead to a different assessment for different raters. Thus, automatic classifier that discriminate SIH patients and healthy controls are currently being developed [23].

**Table 1:** SIH signs on MRI of the head: “Bern” score (18)

Major criteria	
Engorgement of venous sinuses	2
Pachymeningeal enhancement	2
Suprasellar cistern ≤ 4 mm	2
Minor criteria	
Subdural fluid collection	1
Prepontine cistern ≤ 5 mm	1
Mamillopontine distance ≤ 6.5 mm	1
Sum	9
Low risk ≤ 2 points	
Intermediate risk 3–4 points	
High risk ≥ 5 points	

## Spine MRI

As almost all SIH cases are caused by spinal CSF leaks [20, 21], spinal MRI is complementary to head scans. T2-weighted sequences show so-called spinal longitudinal extradural fluid (contrast) (SLEC) in 60% of patients. A protocol with isotropic T2-weighted sequences that allow to display the SLEC (and sometimes even an underlying bony spur) in at least sagittal and axial reformations is sufficient. Note that there are patients without SIH signs on head scans but with spinal CSF leaks, so that with appropriate clinical symptoms, MRI scans of the head and the spine scans should be performed.

Almost all SIH cases are caused by spinal leaks [20, 21]: Around 60% are ventral dural tears typically with tiny bony spurs sticking within a longitudinal cut of the dura (type 1 leak = SLEC-positive) [20]. Around 20% are leaking nerve root sleeves; typically, the leak is in the shoulder of a meningeal diverticulum of the nerve root sleeve (type 2 leak, most often SLEC-positive) [20]. If the dural tear of the nerve root sleeve is distal, there is no CSF within the epidural space (type 2 leak, SLEC-negative or type 4 leak according to Farb, respectively) [20, 21]. Up to 20% of spinal CSF leaks are due to CSF-venous fistulas in which the CSF flows into abnormal venous channels surrounding the nerve root sleeve (type 3 leak = SLEC-negative). The numbers are not fixed as CSF-venous fistulas — initially described in 2014 [24] — are increasingly being identified in recent years.

The further diagnostic workup — after MRI scans of the head and the spine have been acquired — depends on the head and spine findings.

**Fig. 1:**
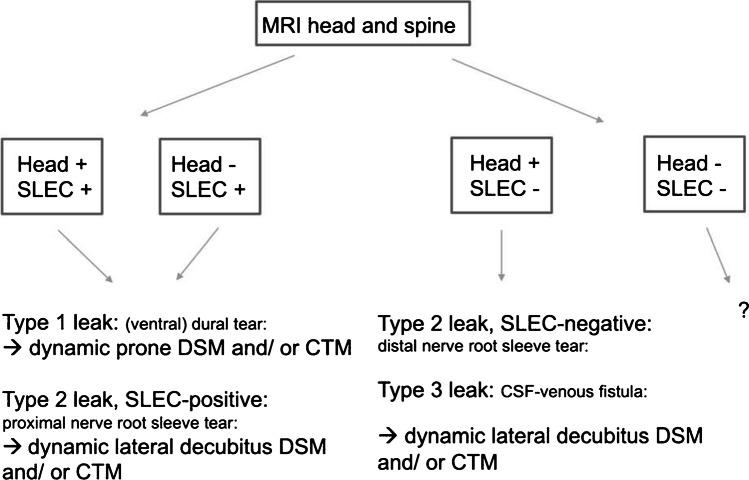
Diagnostic algorithm for patients with suspected SIH

**Fig. 2:**
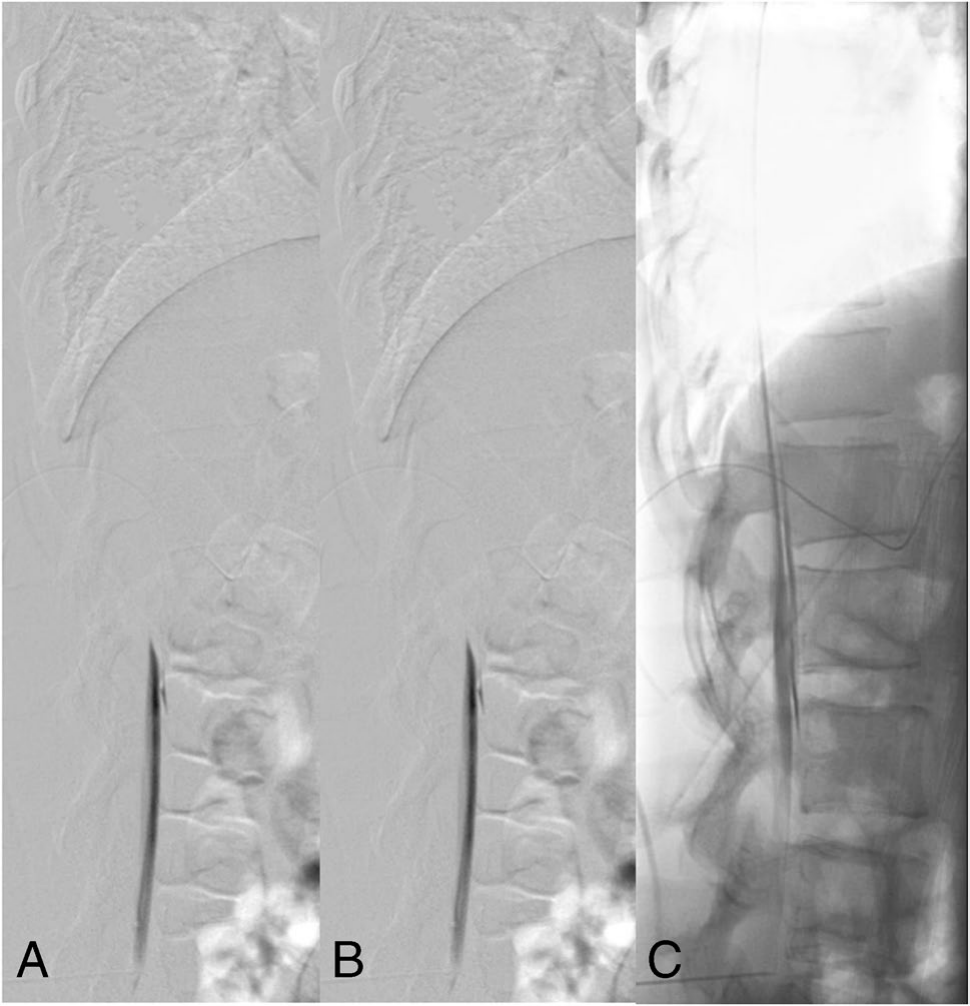
Type 1 leak: Dynamic digital subtraction myelogram (DSM) with the patient in prone position on a tilted table. Due to the higher specific weight compared to CSF, iodinated contrast medium collects at the ventral surface of the thecal sac. With the X-ray tube in lateral and the patient in head down position (around 15°), digital subtraction myelograms are acquired during gentle injection of highly concentrated (300 mg/ml) contrast medium. Using this technique, most of the ventral leaks in the lower thoracic and lumbar spine can be visualized. In this 30-year-old woman, dynamic DSM shows a ventral leak at the level L1/2 (**A**, **B**). Note that on the subsequent unsubtracted fluoroscopic image the exact site of the leak cannot be determined any longer (**C**)

In the so-called head-positive, SLEC-positive and in head-negative, SLEC-positive patients (see Fig. 1), there is high likelihood of a ventral dural tear. Next step is dynamic (digital subtraction) myelography. The patient lies in prone position; the head of the table is tilted down, while lateral fluoroscopic or digital subtraction images are acquired during gentle intrathecal contrast injections [25]. The goal is to locate the ventral dural tear by exactly locating the point of egress of iodinated contrast medium into the epidural space. This is achieved with the iodinated contrast medium slowing flowing into a caudo-cranial direction on the ventral side of the subarachnoid space [25–27] (Fig. 2).

**Fig. 3:**
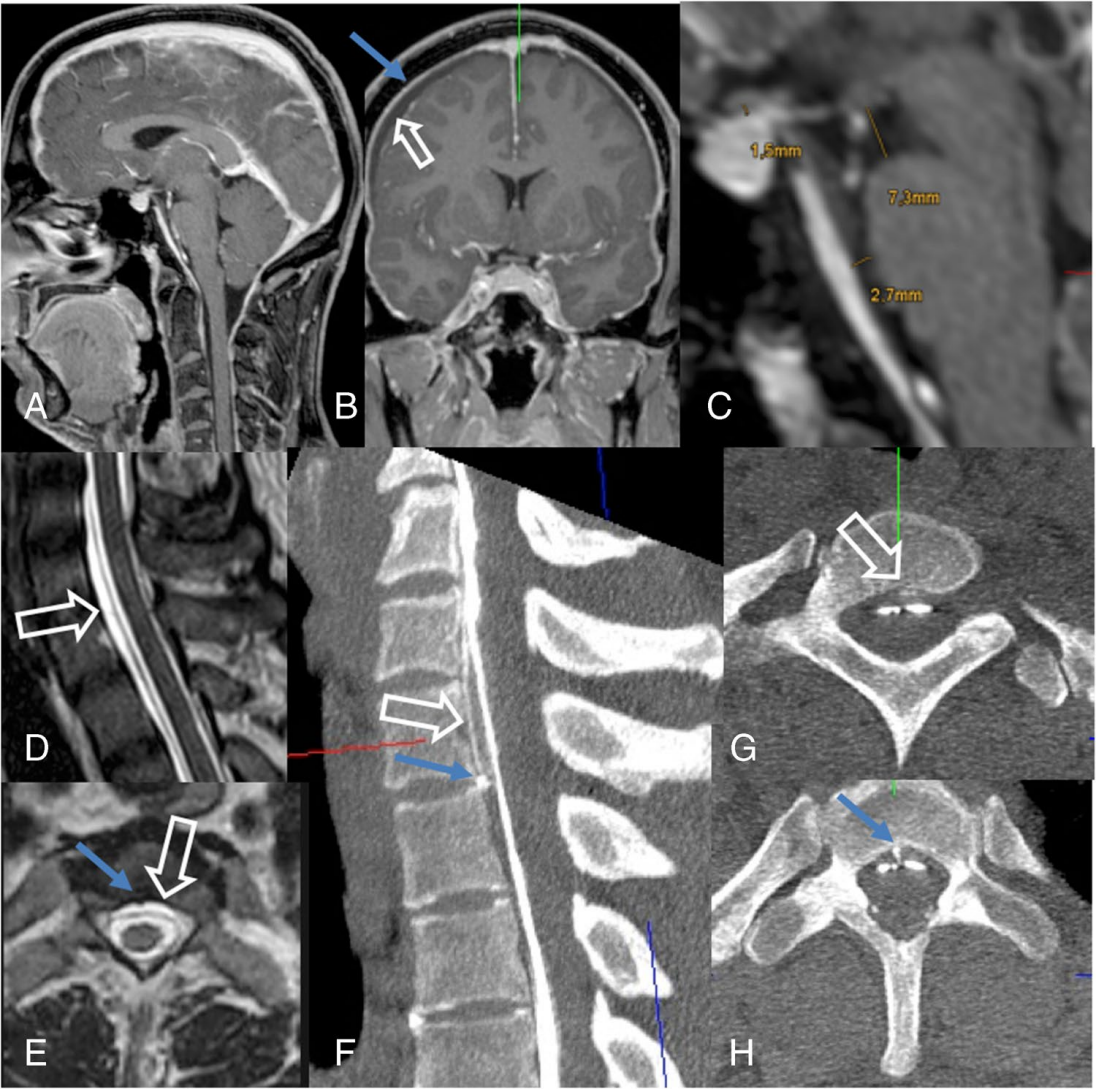
Type 1 leak: SIH in a 36-year-old woman with engorgement of the venous sinuses, pachymeningeal enhancement (**B**: arrow), subdural fluid collections (**B**: hollow arrow), effaced suprasellar (**C**: 1.5 mm), and prepontine cisterns (**C**: 2.7 mm). The mamillopontine distance is normal (**C**: 7.3 mm). Spinal MRI shows spinal longitudinal extradural fluid (SLEC) (**D**, **E**: hollow arrow). Prone dynamic CT myelography shows the ventral leak due to a bony spur (**H**: arrow) and ventral epidural contrast flowing upwards (**F**, **G**: hollow arrow)

Around 50% of ventral dural tears are located in the upper thoracic spine (mostly TH1/2 and Th2/3). Here, it is often difficult to see the ventral leak due to X-ray attenuation of the shoulders. In these situations, we (anticipate to) proceed with prone dynamic CT myelography on another day. However, as the field of view of dynamic CT myelography is limited and radiation exposure is higher, we almost always start with dynamic (digital subtraction) myelography. For dynamic CT myelography, the needle is placed under fluoroscopic control or in the CT scanner into the thecal sac, the patient is positioned on a custom-made, tilted table in the CT scanner, and the upper thoracic region is scanned while the contrast medium is injected. In order to limit the radiation exposure, we follow the ascending contrast column with bolus-tracking and program three spiral scans in caudo-cranial, cranio-caudal, and again caudo-cranial directions (Fig. 3). Radiation exposure, however, is significant and has been reported with 19.7 (3.2–82.4) mSv for dynamic CT myelography compared to 6.6 (1.2–17.7) mSv per DSM study [28].

**Fig. 4:**
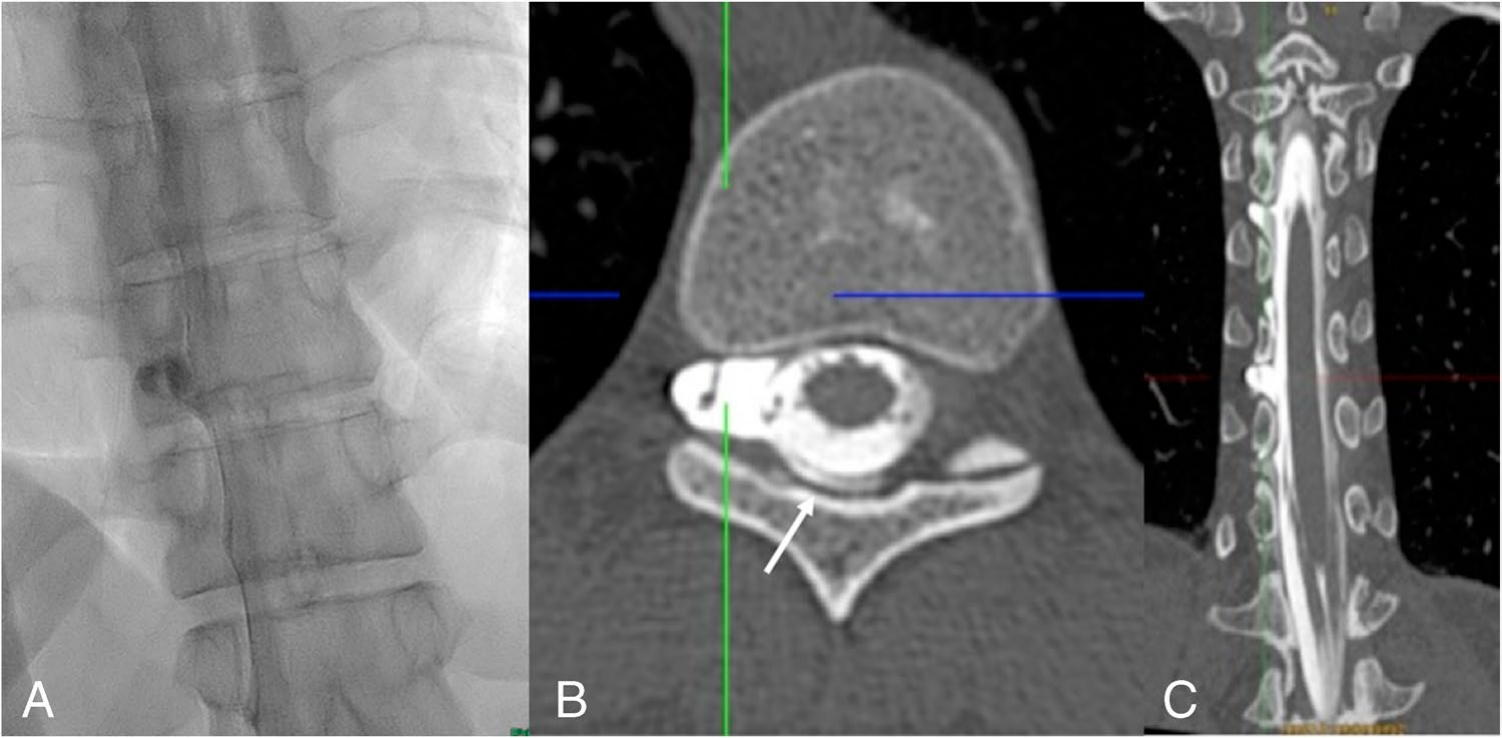
Type 2 leak: Right-sided lateral decubitus myelography shows a leaking meningeal diverticulum Th 10/11 on the right-side (**A**). Subsequent lateral decubitus CT myelography (**B**, **C**) shows extradural contrast (**B**: arrow) indicating a proximal leak

**Fig. 5:**
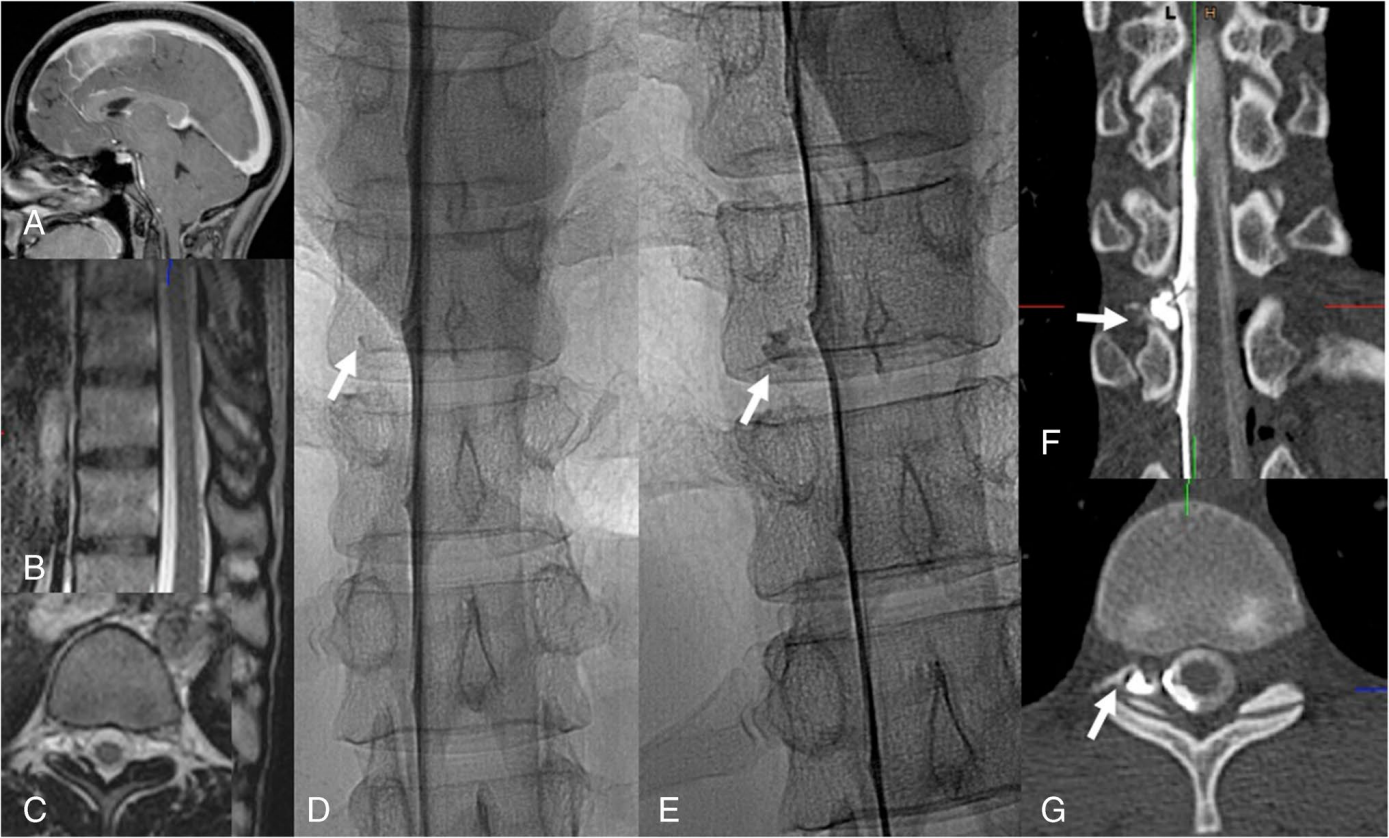
Type 2 leak according to Schievink or type 4 leak according to Farb, respectively: Head-positive (**A**), SLEC-negative MRI scans (**B**, **C**). Right-sided lateral decubitus dynamic myelography shows a slowly filling meningeal diverticulum (**D**, **E**: arrow). Right-sided lateral decubitus dynamic CT myelography shows the extrathecal contrast (**F**: **G**: arrow)

**Fig. 6:**
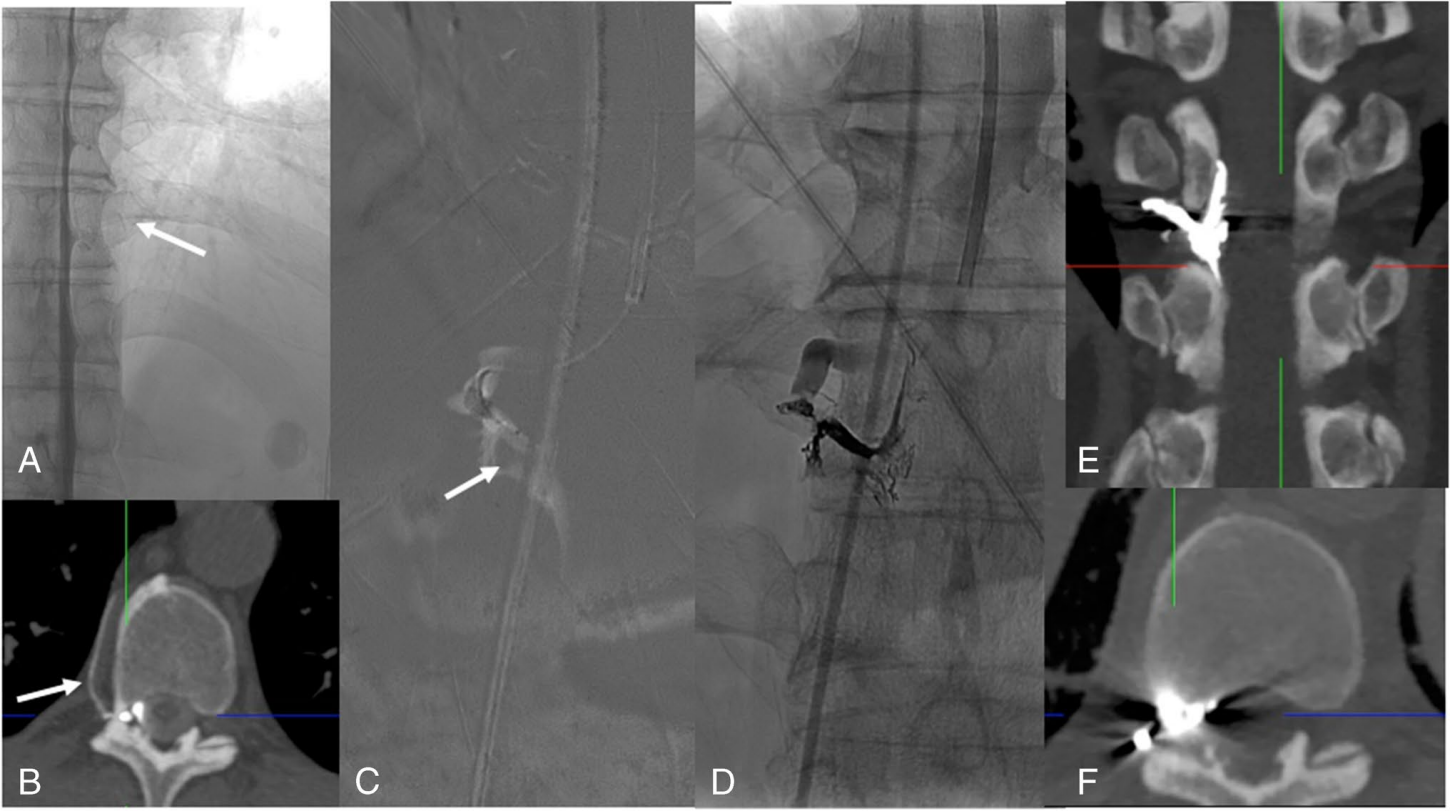
Type 3 leak: Right-sided lateral decubitus dynamic myelography shows a CSF-venous fistula, i.e., here, the tiny venous filling at the level Th 10/11 (**A**: arrow). Right-sided lateral decubitus CT myelography clearly shows a hyperdense paraspinal vein confirming a CSF-venous fistula (**B**: arrow). For transvenous embolization, a Scepter dual‐lumen balloon catheter (Microvention, Tustin, CA, USA) is navigated via the azygos vein into the collector of the veins surrounding the nerve root (**C**: The position of the nerve root is marked with an arrow). Onyx deposition (**D**). Post-embolization CT shows Onyx cast in the desired position (**E**, **F**)

If the SLEC has a lateral and not a ventral extension, it is necessary to demonstrate the leaking nerve root sleeve or diverticulum with lateral decubitus myelography or lateral decubitus CT myelography (Fig. 4). In head-positive, SLEC-negative patients, a CSF leak may be located distally in the nerve root sleeve so that the CSF flows into the fasciae outside the neural foramen (Fig. 5). Another possibility in a head-positive, SLEC-negative patient is the presence of a so-called CSF-venous fistula. Although the CSF is typically filtrated into spinal veins surrounding the nerve root sleeves there may be an abnormal connection — the CSF-venous fistula — which with appropriate intrathecal contrast may be followed as hyperdense structures up to the azygos/hemiazygos vein confluence, the so-called hyperdense paraspinal vein sign [29] (Fig. 6). In order to detect CSF-venous fistulas, it is necessary to acquire fluoroscopic or DSM images in lateral decubitus position, with the hips higher than the shoulders [5–8] and during inspiration. Inspiration results in descent of the diaphragm causing negative intrathoracic pressure as well as increased intraabdominal pressure. This creates a pressure gradient driving blood from the inferior vena cava to the right atrium, increasing venous return to the heart. This blood flow also results in decreased intravascular pressure within the inferior vena cava, which would produce a gradient of pressure between the higher pressure CSF and the lower pressure epidural venous plexus and paraspinal veins [30–33]. CSF-venous fistulas can have a paravertebral (45%), lateral along the neural foramen (23%), or central drainage towards the epidural plexus (32%). Especially for the detection of a CSF-venous fistula, the use of a myelographic contrast media with the highest iodine content allowed (300 mg iodine/ml) is strongly recommended.

Whether it makes sense to search for CSF leaks in head-negative, SLEC-negative patients with appropriate clinical complaints is a matter of debate in SIH centers [34].

Currently, gadolinium myelography is of limited use and of value to locate ventral dural tears and to identify CSF-venous fistulas [35, 36].

## Therapeutic procedures

It is unknown how many patients recover from SIH with (self-directed) bed rest often supplemented with hydration, caffeine, and theophylline (5, 37).

With one or more epidural blood patches, 30–70% of SIH patients show marked improvement; however, a permanent cure in types 1 and 2 leaks is achieved in less than 10% of patients [38, 39]. There is no consensus how to perform a blood patch (“loss of resistance,” fluoroscopy-guided, CT-guided, blood, or fibrin glue) and whether it must be placed precisely over the site of the spinal CSF leak or it might suffice to raise the pressure in the epidural space [40–44]. Punctures at multiple lumbar levels may be needed to deliver the epidural blood to the site of the leak; the technique involves demonstration of an uninterrupted flow of contrast medium (and thus, presumably, of the subsequently injected blood as well) epidurally in the cranial direction. In total, 20–100 mL of blood is injected [38]. Higher volumes are correlated with better therapeutic outcomes [43].

Operative closure of the spinal CSF leak is indicated if the symptoms persist despite less invasive treatment measures and if the spinal CSF leak has been definitively localized [45]. At surgery, the leak can be microsurgically closed with simple suturing, or with an adhesive patch on the dura mater. With current neurosurgical methods, lateral meningeal diverticulae at the existing nerve root, CSF-venous fistulas, and laterally and ventrally located dural tears can be reached through a dorsal approach and closed safely and with minimal invasiveness through an interlaminar fenestration or hemilaminectomy [39, 45]. Ventral dural tears require a transdural approach with detachment of the denticulate ligaments, so that the spinal cord can be mobilized out of the way under intraoperative neuromonitoring [39, 45].

CSF-venous fistulas can be treated with surgical ligation (of the nerve root), with fibrin glue injections into the corresponding neural foramen, or with liquid embolic (Onyx®, eV3 Endovascular, Plymouth, MN) injections into the radicular veins which are catheterized via the route V. femoralis, V. cava superior, V. azygos/hemiazygos [19, 46] (Fig. 6).

Around 1/5 of patients treated with either epidural blood patches or surgery develop symptoms of rebound hypertension (rebound intracranial hypertension RIH) [49]. Patients are usually managed with acetazolamide for some weeks. Risk is higher when treatment is delayed for more than 10 weeks and when patients are obese [16, 49, 50].

## Conclusion

SIH is an increasingly identified CSF disorder in which various imaging modalities play a crucial role for a correct diagnosis and an appropriate therapeutic management.
